# AI-Resolved Protein Energy Landscapes, Electrodynamics, and Fluidic Microcircuits as a Unified Framework for Predicting Neurodegeneration

**DOI:** 10.3390/ijms27020676

**Published:** 2026-01-09

**Authors:** Cosmin Pantu, Alexandru Breazu, Stefan Oprea, Matei Serban, Razvan-Adrian Covache-Busuioc, Octavian Munteanu, Nicolaie Dobrin, Daniel Costea, Lucian Eva

**Affiliations:** 1 Faculty of General Medicine, Carol Davila University of Medicine and Pharmacy, 050474 Bucharest, Romania; 2 University Hospital, Carol Davila University of Medicine and Pharmacy, 010024 Bucharest, Romania; 3 Department of Anatomy, Carol Davila University of Medicine and Pharmacy, 050474 Bucharest, Romania; 4 Department of Neurosurgery, Carol Davila University of Medicine and Pharmacy, 020021 Bucharest, Romania; 5 Department of Vascular Neurosurgery, National Institute of Neurology and Neurovascular Diseases, 077160 Bucharest, Romania; 6 Puls Med Association, 051885 Bucharest, Romania; 7 “Nicolae Oblu” Clinical Hospital, 700309 Iasi, Romania; 8 Department of Neurosurgery, Victor Babes University of Medicine and Pharmacy, 300041 Timisoara, Romania

**Keywords:** neurodegeneration, protein energy landscapes, liquid–liquid phase separation, membrane electrodynamics, dielectric microdomains, neurofluidics, glymphatic clearance, multiphysics coherence, AI-enabled digital twins, operator learning

## Abstract

Research shows that neurodegenerative processes do not develop from a single “broken” biochemistry process; rather, they develop when a complex multi-physics environment gradually loses its ability to stabilize the neuron via a collective action between the protein, ion, field and fluid dynamics of the neuron. The use of new technologies such as quantum-informed molecular simulation (QIMS), dielectric nanoscale mapping, fluid dynamics of the cell, and imaging of perivascular flow are allowing researchers to understand how the collective interactions among proteins, membranes and their electrical properties, along with fluid dynamics within the cell, form a highly interconnected dynamic system. These systems require fine control over the energetic, mechanical and electrical interactions that maintain their coherence. When there is even a small change in the protein conformations, the electric properties of the membrane, or the viscosity of the cell’s interior, it can cause changes in the high dimensional space in which the system operates to lose some of its stabilizing curvature and become prone to instability well before structural pathologies become apparent. AI has allowed researchers to create digital twin models using combined physical data from multiple scales and to predict the trajectory of the neural system toward instability by identifying signs of early deformation. Preliminary studies suggest that deviations in the ergodicity of metabolic–mechanical systems, contraction of dissipative bandwidth, and fragmentation of attractor basins could be indicators of vulnerability. This study will attempt to combine all of the current research into a cohesive view of the role of progressive loss of multi-physics coherence in neurodegenerative disease. Through integration of protein energetics, electrodynamic drift, and hydrodynamic irregularities, as well as predictive modeling utilizing AI, the authors will provide mechanistic insights and discuss potential approaches to early detection, targeted stabilization, and precision-guided interventions based on neurophysics.

## 1. Introduction—Toward a Multiphysics Understanding of Neuronal Failure

The coordination of physical processes that encompass over ten orders of magnitude in both space and time is required for the continued viability of neuronal systems. On one end of the spectrum exist attosecond redistributions of electron density within the side chain of amino acids, while on the other lies millimeter-scale variability of glymphatic flow due to the pulsatile nature of arteries. Between these extremes, neurons are constantly exchanging energy among molecular conformations, gating events of ion channels, membrane electrodynamics, intracellular fluid mechanics, cytoskeletal rheology, astrocytic ion buffering, mitochondrial redox gradients, and perivascular pressure-flow coupling [1]. Physical domains interact via feedback loops that although potentially subtle individually become important if they continue to be disrupted over time. It has been observed that an increasing number of reports indicate that neurodegenerative diseases occur as a result of decoupling among the various physical domains involved in these multiscale systems [2].

There have been recent advancements in the study of structural and computational chemistry concerning the characterization of the conformational ensembles of neuronal proteins. Cryo-electron microscopy with high resolution, beta relaxation spectroscopy, hydrogen-deuterium exchange mass spectrometry and accelerating quantum/molecular-dynamic simulations have demonstrated that proteins like tau, alpha synuclein, TDP-43, CAMK II, PSD-95, Homer I, SynGap, gephyrin and the GluN1/GluN2 subunit of NMDA receptor are present in numerous microstates and are separated by barriers ranging from approximately 2–15 kJ/mol [3]. Machine learned force fields such as ANI-2x, AIMNet, PhysNet, Qingyun, and MACE have allowed for the creation of more accurate representations of the chemical bonding and non-bonding interactions of proteins which allow for a better representation of local minimum energy in the potential energy surface of proteins. This includes shallow metastable wells that could be preferentially occupied by proteins under slight changes in pH, ionic strength, redox status, or post translational modification such as hyperphosphorylation, SUMOylation or ubiquitination [4]. For example, changing the protonation state of a histidine or lysine residue within the repeat domains of tau or the solvation shell surrounding positions E46 or H50 of alpha synuclein can lead to changes in the energy landscape of proteins that can affect LLPS propensity, fibril nucleation, and interactions with microtubules. These molecular level changes affect the electrodynamic domain. The excitability of neurons does not only depend upon the distribution of voltage gated channels such as Nav1.1–Nav1.9, Kv1.x–Kv7.x, Cav1.x–Cav3.x, and HCN1–HCN4 but also upon the free-energy profiles controlling their transition between open and closed states [5]. The classical Hodgkin–Huxley formulation provides a macroscopic description of the transition between the activated and inactivated states of voltage gated channels; however, it is now possible to measure the microscopic energy surfaces controlling these processes. Accelerated MD simulations enhanced using AI, and Markov state models have started to determine how changes in the local dielectric environment, lipid composition and membrane curvature can affect gating kinetics [6]. For example, changes in the amount of cholesterol or the ratio of phosphatidylcholine to sphingomyelin in the membrane can affect dielectric constant values, capacitive reactance and dipole fluctuations which in turn can affect the effective charge displaced (z) during the transition of a voltage sensing domain between its resting and activated states. Such changes can cause changes in the voltage dependence of the open probability of a channel. Over the course of many years such changes can affect conduction velocity, spike timing accuracy, and/or facilitate ephaptic interference [7].

Membrane electrodynamics is also dependent on the interactions among lipid microdomains, cytoskeletal anchoring and protein crowding. Different lipids such as phosphatidylserine, phosphatidylinositol 4,5-bisphosphate (PI(4,5)P_2_), monosialotetrahexosylganglioside (GM1) ganglioside, and cardiolipin produce dynamic dielectric microenvironments that can control local field propagation. Proteins that bind to lipids such as myristoylated alanine-rich C-kinase substrate (MARCKS), Annexins, and Bin/amphiphysin/Rvs (BAR) domain scaffold proteins can shape local curvature fields and change the spatial distribution of surface charges affecting Debye screening length [8]. Collectively, the interactions among lipids, proteins, and the cytoskeleton can control the spatio-temporal behavior of nanoscale electric fields, which are increasingly recognized as regulators of vesicle docking, soluble N-ethylmaleimide-sensitive factor attachment protein receptor (SNARE) complex assembly, and synaptic transmission fidelity. Fluid mechanics and transport phenomena create a further layer of physical regulation in addition to those of intracellular and extracellular ion transport. The neuronal cytoplasm functions as a concentration-dependent viscoelastic medium whose mechanical properties vary throughout the cell [9]. FRAP, single particle tracking and active microrheology studies have indicated that the diffusivity of large macromolecules in dendrites can vary by more than an order of magnitude between dendrite shafts and spine heads. Factors contributing to differences in diffusivity include actin polymerization dynamics, cross-linking by proteins such as α-actinin and drebrin, and the formation of phase-separated liquid–liquid phase separation (LLPS) condensates containing RNA-binding proteins such as fused in sarcoma (FUS) or heterogeneous nuclear ribonucleoprotein A1 (hnRNPA1) [10]. Localized decreases in ATP availability can decrease the activity of myosin and therefore decrease the fluidity of the cytoplasm. Additionally, the function of mitochondria can generate localized thermal gradients, produce protons near complex IV, and increase the oxidative stress-induced changes in microviscosity. These varying physical parameters can regulate the transport of metabolites, calcium ions, vesicles, and cytoskeletal components [11].

On the larger scale, the motion of extracellular fluid is regulated by astrocytes with aquaporin-4 (AQP4)4-containing perivascular end-feet, Kir4.1 channels, Connexin-43 gap junctions, and the laminin/collagen matrix of the basement membranes. Perivascular spaces generated by the branching of penetrating arterioles and the venous outflow pathway regulate hydraulic resistance and solute clearance. Recent studies using Navier–Stokes simulations and two-photon imaging have documented transitions between laminar and turbulent flow regimes in these confined spaces [12]. Variations in the compliance of veins, the pulsatility of arteries, the gradients of perivascular pressure, and the localization of AQP4 have been found to regulate clearance efficiency of solutes such as lactate, tau peptides, and amyloid-beta species. The accumulation of such substances can alter osmotic gradients, membrane surface tension, and local electrolyte composition in ways that can affect the stability of proteins and the excitability of neurons [13,14]. Feedback among these physical domains creates a highly coupled system. For example, changes in the populations of microstates in proteins such as tau or alpha synuclein can affect ion-channel gating, which can affect membrane potential dynamics and localized heating, which can affect fluid viscosity and solute transport. Decreases in the clearance efficiency caused by changes in the perivascular flow can change the composition of the extracellular ionic environment, which can affect Nernst potentials, conductance noise, and the voltage dependence of conformational transitions in membrane proteins [15]. Alterations in the viscosity of the cytoplasm can change the rate of diffusion of ATP, influence the cycles of fusion/fission of mitochondria mediated by dynamin-related protein 1 (Drp1; DNM1L) and mitofusin 2 (Mfn2; MFN2), and thus affect the redox gradient that controls the folding energy of proteins. These feedback loops can create a situation where a small perturbation in one domain can cascade through other domains over years or decades [16].

Artificial Intelligence and Machine Learned Models of Physical Systems have recently enabled researchers to begin to understand the interconnectedness of these physical domains. Examples of models of the interactions among these physical domains include physics informed neural networks, operator learning architectures such as DeepONets and Fourier Neural Operators, Quantum Machine Learning Potentials, and Hybrid PDE-AI Solvers. These types of models enable researchers to simulate protein conformational ensembles, membrane field propagation, and fluid/ion transport without simulating each process at atomic resolution [17]. Ultimately, researchers hope that digital twin frameworks that combine these models will allow them to develop an approximation of neuronal dynamics across multiple physical domains and identify early signs of instability. When developing this manuscript an attempt was made to integrate results from quantum chemistry, molecular dynamics, electrodynamics, fluid dynamics, and computational neuroscience [18]. The integration represents an attempt to find common ground across disciplines rather than to exhaustively review each discipline. The intent of the manuscript is to establish a theoretical framework that views the energy landscape of proteins, the behavior of electrical fields, and the fluidic micro-circuits as parts of a unified system whose coupled stability affects susceptibility to neurodegenerative disease pathways.

The literature search for each part of this paper was performed through a series of iterative searches which included various combinations of specific-disease/mechanism related keyword searches and broad terms that would produce large numbers of references that are potentially useful. Literature specifically concerning diseases in this search include: tau, alpha-synuclein, TDP-43, IDPs (intrinsically disordered proteins), LLPS (biomolecular condensation), cryo-EM dynamics, HDX-MS, dielectric and beta relaxation spectroscopy, molecular dynamic simulations (MD), MSMD (Markov State Model), free-energy landscapes, machine learned force fields (e.g., ANI, AIMNet, PhysNet, MACE), domains of voltage gated ion channels involved in gating, dielectric environments, lipid micro-domains, membrane curvature, ephaptic interactions, viscosity/rheology of the cytoplasm (FRAP, SPT, active microrheology), oxidative stress/redox of mitochondria, and glymphatic flow/perivascular flow of fluid (AQP4, CSF-ISF exchange, two photon imaging, Navier–Stokes equations, laminar–turbulent transition), as well as artificial intelligence (AI)-based multiphysics modeling (PINNs, operator learning: DeepONets/Fourier Neural Operators, hybrid PDE-AI solvers/physics-informed neural network). Where appropriate, these areas of study were connected to Alzheimer’s disease, Parkinson’s disease, frontotemporal dementia (FTD), and amyotrophic lateral sclerosis (ALS). No AI model was used to generate any manuscript content.

After identifying title and abstracts, screening was conducted to identify relevance to at least one of the focused domains: protein conformational energetics, membrane electrodynamics, intracellular rheology/transport, perivascular hydrodynamics/glymphatic clearance, and AI-based multiphysics modeling. In addition to screening full-text articles for relevance, assessments of methodological rigor were also evaluated including (i) experimentally verified and quantitatively measured data, (ii) computationally reproducible protocols and methods, and (iii) quantitative modeling of biological systems. Evaluations of full-text articles also identified whether there was evidence of coupling and/or decoupling between biological domains. The highest priority was given to primary mechanistic studies, articles describing rigorous methodologies, and articles describing integrated multi-scale studies. Due to the rapid development of glymphatic clearance studies, the most recent literature available (approximately 5–10 years) was given the highest preference when available; however, foundational studies that established key concepts were also retained.

Studies that lacked specificity towards mechanisms of interest, studies containing mostly speculative information without providing supporting data, redundant studies relative to more robust literature, or studies that lacked direct information toward the proposed unifying mechanism, were either excluded or de-prioritized.

This narrative paper is intended for cross-disciplinary researchers spanning neuroscience, biophysics, computational modeling, and machine learning who seek a unified multiphysics framework for neurodegeneration. To maintain scientific balance, the manuscript is written with a deliberately cautious tone, distinguishing descriptive synthesis of the existing literature from broader interpretive statements. Where appropriate, integrative concepts are presented as a perspective intended to encourage further discussion and research rather than as definitive conclusions.

The subsequent sections describe this system step-by-step. Section 2 reviews the chemistry and physics of protein energy landscapes, specifically conformational ensembles and their perturbed transitions. Section 3 examines electrodynamic processes and how their coherence can deteriorate. Section 4 evaluates intracellular and perivascular fluid dynamics. Section 5 combines the above-mentioned processes into a multiphysics failure model. Section 6 discusses AI-assisted multiphysics digital twins. Section 7 addresses their potential use for prediction and intervention. Section 8 closes with thoughts on potential future directions.

## 2. Protein Energy Landscapes as Early Determinants of Neuronal Destabilization

### 2.1. AI-Based Reconstruction of Neuronal Protein Energy Landscapes

Quantum chemistry data used to create neural network potentials (Accurate NeurAl networK engINe (ANI)-style, equivariant message passing, etc.) allow researchers to model the free energy surface of various proteins that play important roles in both normal neuronal function and neurodegenerative diseases [19]. Researchers have created models of tau’s repeat domains (R1–R4), α-synuclein’s N-terminal alpha-helix and C-terminal non-alpha helix regions (NAC), and low complexity segments of TAR DNA-binding protein 43 (TDP-43; TARDBP), fused in sarcoma (FUS), and heterogeneous nuclear ribonucleoprotein A1 (hnRNPA1) using a variety of enhanced sampling methods (parallel tempering, replica exchange, metadynamics, and variational bias) [20]. Models of tau’s hexapeptides, e.g., tau hexapeptide motifs VQIINK (residues 275–280) and VQIVYK (residues 306–311), show that these peptides will populate multiple basins on the free energy surface, and that the relative energy of these basins will depend on the protonation of lysines and histidines, the distribution of counter ions (chloride, phosphate), and the variation in solvation. Models of α-synuclein show that residues at positions 61, 66, 74, and 76 form hydrophobic microclusters that come into being and disappear based on changes in the backbone torsions [21]. Models of the low complexity domains of TDP-43 and FUS show that simulations can detect transient π–π and cation–π interactions among tyrosines, phenylalanines, and arginines, and find clusters of side chains whose cooperativity is affected by phosphorylation, methylation, or binding to uridine rich RNA sequences. Markov State Models of these trajectories show that there are many slowly interconverting basins of microstates on the free energy surface of these proteins on timescales of microseconds to milliseconds, and that the rates of transition between these basins are related to the height of the energy barriers separating them, which are often less than 10 kcal/mol [22]. Thus, even relatively small energy barrier reductions caused by environmental changes in temperature, pressure, or ionic strength can significantly alter the populations of the microstates, without causing large changes in the structure of the protein. Models of the free energy surfaces of synaptic scaffold proteins such as PSD-95/Discs large/ZO-1 (PDZ), Shank, Homer, and SynGap show that they have many interdomain orientations that affect the accessibility of the PDZ grooves, SH3 binding pockets, and guanylate kinase (GK) interaction surfaces of these proteins. These orientations can be changed by the phosphorylation (e.g., by CaMKII dependent sites), palmitoylation of PDZ cysteines, or local pH changes caused by synaptic activity [23].

Physically, the free energy surfaces of these proteins can be modeled as functions F(q) that depend on a set of collective coordinates q, such as backbone hydrogen bond metrics, side chain dihedral angles, interresidue distances, and/or radius of gyration parameters. Neural potentials provide an approximation of the potential energy U(q) associated with these coordinates [24]. Solvent and entropy contributions are typically obtained through molecular dynamics. Many neuronal proteins have very rough free energy surfaces, indicating that the local minima have similar energies, making it easy for minor changes in ionic strength, redox state, or solvent polarization to change the populations of the microstates [25].

Because many functional states exist near each other on the free energy surface, i.e., within a range of approximately 10 kBT, neuronal proteins require precise regulation of the constraints to maintain the desired population distributions of the microstates. Proteins called chaperones (Hsp70, Hsp90), co-chaperones (DnaJC family), and controlled cycles of phosphorylation and dephosphorylation are all able to regulate these constraints [26]. However, when neurons experience oxidative or energetic stress, changes in the regulatory inputs can lead to changes in the shape of the free energy surface of the proteins. For example, oxidative modifications of methionines or cysteines, dityrosine crosslinking, or glycation of lysines can alter the packing of the amino acids. Changes in solvation caused by an osmolyte imbalance or an ion redistribution can also alter the hydrogen bonding network between the amino acids, changing the barrier heights and the curvature of the free energy surface [27].

### 2.2. Phase Behavior, Liquid–Liquid Phase Separation, and Mesoscale Proteome Organization

Many neuronal proteins undergo liquid–liquid phase separation, forming condensates whose physical and dynamic properties are determined by multivalent interactions among their intrinsically disordered regions. Intrinsically disordered regions commonly have “sticker” motifs (aromatic residues, arginines, acidic or basic clusters, proline-rich motifs) separated by flexible spacers [28]. Associative polymer models explain the emergent behavior of these regions in terms of the valence of the stickers, their spatial distribution, their interaction energies, and the influence of the quality of the solvent. Simulations of low-complexity domains of TDP-43, FUS, and hnRNPA1 showed that π–π interactions among tyrosines and phenylalanines and cation–π contacts between arginines and aromatic rings drive droplet formation [29]. Coarse grained simulations and experimental measurements of saturation concentrations were used to construct phase diagrams showing the conditions under which a single phase or two phases exist, and the location of the critical point, which depends on phosphorylation, arginine methylation, adenosine triphosphate (ATP) levels, or ribonucleic acid (RNA) concentration. Synaptic condensates formed by PDZ, Shank, Homer, and SynGap exhibit analogous principles. PDZ–ligand interactions, Src homology 3 (SH3)–proline-rich contacts, and sterile alpha motif (SAM) domain oligomerization generate modular network architectures whose phase behavior is affected by CaMKII autophosphorylation or Ca^2+^/calmodulin binding [30,31]. In pre-synaptic terminals, condensates involved in the vesicle release machinery include multi-valent interactions among Rab3-interacting molecule (RIM), ELKS, Munc13, and α-liprins. Membrane lipids such as PI(4,5)P_2_, cholesterol, and GM1 affect the stability of the condensates by affecting the local electrostatics, interfacial tension, and effective concentration of the scaffold proteins [32].

Inside the condensates, the concentration of the proteins can increase by up to two orders of magnitude, which affects the kinetic rate of reactions through mass action effects, decreases translational diffusion, increases viscosity, and changes the structuring of the solvent. Condensates can also have internal heterogeneities, with the core and shell having different mobilities and compositions. Some droplets can age, during which they go from liquid-like to gel-like states, and this process involves changes in the sticker–spacer interactions, the formation of quasi-ordered cross-β motifs, or the changes in the RNA-binding dynamics [33]. In stressed neuronal systems, condensates can accumulate oxidized residues, altered charge distributions, or partial dehydration, and this can change their mechanical and dynamic properties. Gradients of composition at interfaces between condensates and surrounding cytoplasm can influence protonation equilibria, ion partitioning, and interaction energies [33]. These gradients can affect the local hydrogen-bonding networks, and thereby weaken or strengthen the interactions. Interfacial tension determines whether droplets will coalesce or undergo Ostwald ripening. Droplet–membrane interactions in crowded synaptic regions can cause curvature-dependent partitioning of proteins and lipids, which can influence the local electrodynamics and mechanical properties [34].

Thus, the proteome of a neuron can be viewed as a high dimensional phase space, with each dimension corresponding to variables like condensate valence, interaction strength, concentration, ionic composition, redox state, and ATP/ADP ratio. Small changes in any of these dimensions can shift the neuron across phase boundaries. These shifts do not necessarily result in fibrillar aggregates; rather, intermediate states characterized by altered droplet turnover, increased gel fraction, or minor changes in microscopic viscosity can be indicative of early deviation from homeostasis [35]. The following Table 1 provides a summary of the multiscale organization of the neuronal protein energy landscapes, illustrating how quantum level perturbations can be propagated through molecular, meso-scale and cellular systems to induce early physicochemical instability. This Table 1 integrates the structural information generated by the use of artificial intelligence, with the biophysical and phase behavior principles described above to illustrate how minor energy barrier reductions can occur before obvious aggregation or neurodegeneration.

### 2.3. Energy-Barrier Erosion and Early Physicochemical Signatures of Instability

While most researchers have focused on major structural alterations to proteins due to their destabilized energy landscapes, smaller alterations to multiple proteins can lead to early signs of instability when their energy landscapes undergo minor changes. This process can be characterized using various physical chemistry metrics; specifically barrier height, curvature of the energy landscape near both the minimum and the saddle point of the protein’s conformational space and frictional forces affecting the protein’s diffusive motion [41]. Since these frictional forces are directly related to the protein’s diffusive motion, reducing them can enhance the speed of transition between basins of attraction of the protein’s energy landscape. Oxidative modification, methionine sulfoxide and cysteine oxidation, glycation, nitration, lipid peroxide products that bind to proteins, and increased concentrations of macromolecules can produce these types of frictional force reductions in neurons [42]. Changes to local environment, including pH, redox potential, salt concentrations, and concentrations of osmolytes also alter the solvation shell and hydrogen bonding and therefore alter the dielectric constant and viscosity around each protein [43]. As such, they modify the pre-factor term in rate equations through the alteration of the local curvature of the potential energy surface of the protein [44].

Regions of the energy landscape with decreased curvature allow for greater fluctuations in structural parameters while regions with increased curvature, particularly those that relate to functional motions of the protein (such as ligand exchange loops, domain rotation, and hinge bending) can slow down the necessary transitions required for the proper functioning of the protein [45]. Similar mechanisms are operative in liquid–liquid phase separation systems where slight decreases in the energy penalty for chains to move and rearrange themselves result in an increased probability of β-sheet nucleation in droplets. Increased density environments reduce the entropic cost associated with the alignment of aggregation prone segments of protein sequences [46].

Similarly, the same type of mechanism exists in actin and microtubule systems where changes in the state of nucleotides, cation binding (Mg^2+^, Ca^2+^), and post translational modifications (acetylation, detyrosination) impact the free energy of polymerization and the stability of filaments. Similarly, in mitochondrial proteins, changes in the protonation state of residues or the transmembrane potential can impact the conformational equilibrium of protein–protein interactions within electron transport chain complexes impacting the amount of reactive oxygen species (ROS) produced and the heat generated, which can feedback to the local stability of proteins via thermal fluctuations [47]. Collectively these changes can manifest as changes in relaxation time, fluctuation amplitude, and transition path time, and can be observable through changes in nuclear magnetic resonance (NMR) relaxation dispersion profiles, the persistence of partially unfolded intermediates observed in long timescale simulations, and changes in fluorescence lifetime distribution measured in single molecule experiments. While it is still difficult to measure these changes directly in neurons, these can be inferred based on AI analysis of MD simulation data and experimental perturbation data [48].

Summary Section: Proteins may become vulnerable to neuronal injury due to a shift in their free energy landscape; this can be caused by relatively small physico-chemical changes (pH, salt concentration, redox environment, or post translational modifications). The relative position of states along the free energy surface may remain the same but the relative proportions of states will change and these changes can occur over extended periods of time, and therefore may lead to subtle alterations in the barrier height and/or curvature of the free energy landscape. This can cause the protein’s equilibrium and phase behavior to be altered and create conditions for nucleation over extended periods of time. It is hypothesized that such small changes can serve as an initial “instability axis” through which other types of instability can be propagated via electrodynamic and hydrodynamic coupling that are examined in detail in the next section regarding long term neuronal stability.

## 3. Electrodynamics of the Diseased Neuron

The molecular fluctuations we have discussed above will also manifest at the ionic and electrodynamic levels of the neuron due to the interaction between voltage-dependent proteins, charged lipids and the fluctuating ionic cloud to produce a coherent electrical signal, which is ultimately dependent upon their interaction with each other, the thermodynamic forces driving them and the physical laws governing their motion [49].

Additionally, the macroscopic electrodynamic behavior of the cell will be influenced by molecular ensemble fluctuations at a stage prior to observable structural deterioration of the cell [50].

### 3.1. Nanoscale Ionic Thermodynamics and Evolving Dielectric Microdomains

The fluctuations in the energy landscapes of proteins that we have discussed above translate into the ionic and electrostatic environments responsible for generating the resting membrane potential of the neuron. Studies carried out at the nanoscale reveal that neuronal membranes act as composite dielectric landscapes composed of various domains characterized by distinct dielectric constants generated from phosphatidylserine-rich domains, PI(4,5)P_2_-rich nanodomains, cholesterol-stabilized rafts, GM1 microdomains, and polyunsaturated phospholipid-rich regions with distinctly different dipole moments and polarizations [51]. As a result, each domain influences the electric potential according to a Poisson equation describing the spatial gradient of the electric potential: ∇⋅[εr(r)∇ϕ(r)] = −ρ(r).

Recently, molecular simulations powered by AI-driven potential predictors based on transformer architectures and neural-QM approximators demonstrated that voltage-sensor helices interact with the nanoscale rearrangement of lipid tails to modify their hydration energies and microionic polarization [52,53]. Furthermore, they discovered previously undetermined interactions between charged S4 residues and the dynamic changes in lipid dipoles indicating that relatively minor changes in lipid composition can influence the energetic demands required for the opening of the channel. Fluctuations in ions contribute to further electrodynamic instabilities. Reaction–diffusion solvers enhanced by AI using Physics-Informed Neural Networks (PINNs) showed that Ca^2+^ microdomains located near Cav2 channels show both anisotropy and energy dependency and that the pathways of diffusion are influenced by ATP-driven flows in the cytoskeleton [54]. The models illustrated how slight changes in the affinity of buffer proteins or the proton gradients produced by mitochondria would dramatically alter the distribution of Ca^2+^ in ways that propagate to the probability of vesicle release and to short-term synaptic plasticity. The Na^+^/K^+^-ATPase, V-type H^+^-pumps, and mitochondrial Na^+^/Ca^2+^ exchangers function as local energetic integrators whose rates of turn-over connect the metabolic state of the cell to the membrane potential of the cell [55]. Studies of single molecules revealed that the hydrophobic mismatch between proteins and the lipid bilayer influences the energy of the pump and connects the remodeling of the membrane to the stability of the neuronal voltage [56].

### 3.2. Electromechanical Field Propagation, Resonance Drift, and Dielectric Heterogeneity

Signal transmission in neurons occurs over long ranges through the cooperative actions of ion movements, membrane rigidity, lipid composition, and distributed protein states. The capacitance of the membrane is no longer considered a fixed value; rather it varies with the curvature and packing of lipids to produce micro-domains where electric fields are locally focused or damped [57]. Using deep learning-based dielectric tomography, deep neural-field solvers have shown that the time-varying polarization fields that affect the kinetics of channels through electromechanical coupling are produced by gradients of curvature in structures, including the neck of a dendritic spine [58].

The resonance of sub-threshold type produced by HCN, Kv7, and T-type Ca^2+^ channels require specific energetic communication among cyclic nucleotide-binding domains, voltage-sensing domains, and membrane lipids. Recent structures and refined free energy surfaces produced by AI using cryo-EM demonstrate that resonance behavior is very sensitive to small biochemical drifts, such as mild oxidation or changes in the availability of PI(4,5)P_2_ [59]. These drifts result in substantial changes to the tuning of theta- or beta-band resonance throughout hippocampal and cortical networks. Transformer-based gate-energy predictors that were trained on MD trajectories have demonstrated that channel clusters exist in metastable configurations that establish frequency selectivity in ways that cannot be modeled using classical electrophysiology models. Similarly, conduction velocities along axons are also influenced [60]. Paranodal junctions formed from contactin-1, Caspr, and neurofascin-155 function as microscopic electrical compartments that vary depending on the dielectric composition of compact myelin. Models of dielectric spectroscopy that are enhanced by AI predict that compact myelin is a frequency-dispersive material; the permittivity of compact myelin decreases with increasing frequency as a result of constraints imposed by lipid-tail ordering [61]. Only using advanced mass spectrometry can detect small changes in sulfatide or ceramide content that can affect conduction velocity and spike timing accuracy. In addition, nanometer-scale temperature gradients caused by mitochondrial clusters induce gating kinetic effects via Q10 effects, producing spatially non-uniform electrodynamic responses. Gradual molecular perturbations in the membrane of the neuron propagate to the larger ionic and electrodynamic domains, influencing how fields propagate, how resonance is tuned, and how coherence is maintained across neuronal networks [62,63].

**Figure 1:**
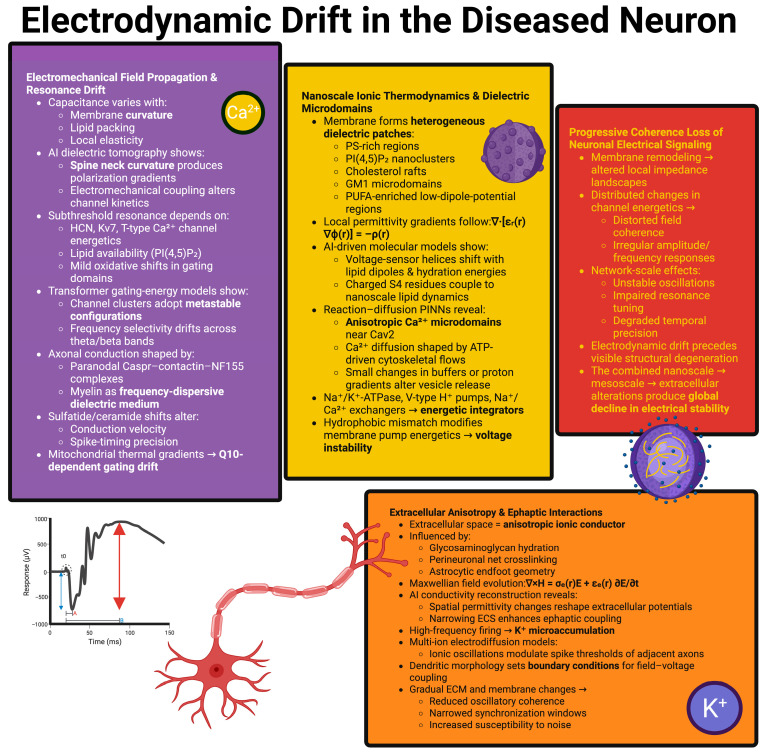
Electrodynamic Drift in the Diseased Neuron. This Figure demonstrates the molecular scale deformation of the protein energy landscape leading to a gradual progression of instability within all of the domains of the neuron’s electro-dynamic properties including dielectric, electrolytic, mechanical–electromechanical and external to the cell membrane.

Figure 1 illustrates this sequence of destabilizing neuronal electrical behavior at multiple scales and shows how dielectric shifts at the nanoscale, electromechanical coupling, and extracellular anisotropy contribute to the destabilization of neuronal electrical behavior.

### 3.3. Extracellular Anisotropy, Ephaptic Interactions, and Progressive Coherence Loss

The extracellular space serves as an anisotropic conductor whose electrodynamic properties are modified by the neuronal activity, metabolic condition, and age of the neuron. Nanoimpedance measurements and AI-generated conductivity maps have been used to demonstrate that the extracellular conductivity and permittivity are significantly varied by differences in the hydration of glycosaminoglycans, perineuronal net cross-linking, and the geometry of the astrocyte end-foot. These variations affect the propagation of extracellular potentials and modulate ephaptic interactions [64,65,66].

In extracellular tissue, electric fields satisfy Maxwell’s equations with spatially inhomogeneous conductivity: ∇ × H = σe(r)E + εe(r)∂t/∂E.

Physics-informed neural networks trained on high-density electrophysiological recordings have provided accurate models of the evolution of extracellular fields with previously unknown precision, illustrating that small changes in matrix composition can significantly affect the coherence of extracellular fields over large spatial scales. Narrowing of the extracellular space due to astrocytic swelling or osmotic fluctuations enhance ephaptic interactions [67]. High-frequency firing causes localized K^+^ accumulation, and recent electrodiffusion models using learned nonlinear diffusion operators demonstrate that these ionic fluctuations can modify the threshold of spike generation in adjacent axons. Variations in the morphology of the dendrites affect the boundary conditions that control how the extracellular fields communicate with the intracellular voltage [68,69]. Graph neural network integrated multi-scale finite element reconstructions have shown that variations in membrane conductivity or branching of the dendrites will redistribute impedance landscapes, affecting how neurons filter extracellular signals. These cumulative changes occur as the membrane composition changes, the extracellular anisotropy increases, and the ionic dynamics fluctuates. As a result, there is a progressive narrowing of synchronization windows, reduced stability of oscillation patterns, and increased susceptibility of neuronal networks to noise [70].

Summary Section: This Section discusses how the electrodynamics of the plasma membrane are controlled by factors other than just the number of ion channel proteins embedded within the membrane (as discussed previously). The authors also discuss how the electro-dynamics of the plasma membrane are affected by: (1) free-energy dependent opening/closing kinetics of the gates of individual ion-channel protein subunits; (2) the dielectric properties of the immediate environment of each domain; (3) the formation of small, localized lipid micro-domains within the bilayer; and (4) the formation of domains that are anchored to the cytoskeleton. These four factors work in concert to determine the ability of an electrical field to propagate across the membrane and also affect the ability of a neuron to generate resonance at specific frequencies. A small or significant alteration in membrane chemistry or the local screening of ions, either short-term or over longer periods of time, can lead to significant changes in the voltage dependence, time-keeping and overall coherence of the electrical networks of neurons. This may result in the sensitivity of the neuron to external interferences, which could potentially cause an excitability drift. The authors show that the study links the micro-conformational energetics of the ion-channel proteins to macroscopic voltage stability and demonstrates electro-dynamic coherence as a second, coupled axis of stability to the multiphysics failure model.

## 4. Fluidic Microcircuits and Hydrodynamic Instabilities Across Intracellular and Perivascular Domains

### 4.1. Quantum-Informed Intracellular Hydrodynamics and Viscoelastic Transport

The fluid behavior in the neurons is based on the intra-cellular environment being a dynamically organized visco-elastic fluid, rather than simply an aqueous solution. Nano-confined water networks are formed within the cell due to confinement caused by cytoskeletal filaments, organelles, and other large macromolecules, changing the hydrogen bonding between the confined water and the surrounding macromolecules [71]. Machine learned potentials supported by quantum mechanical simulations have demonstrated that nano-confined hydration layers exhibit partial proton delocalization, creating short lived proton conduction chains that fluctuate with mitochondrial redox micro-gradients and influence the protonation equilibria of charged amino acids near voltage sensing helices, thus connecting quantum proton mobility to macroscopic electro-physiology. High speed particle imaging has demonstrated that the intra-cellular fluid behaves as a weakly shear thinning fluid [72]. Relaxation times in the intra-cellular fluid are dependent upon ATP dependent turnover of cross-linking proteins such as filamin, alpha-actinin, and fascin. PINNs (physics informed neural networks) were used to train on intra-cellular particle trajectories, generating anisotropic velocity fields, showing preferred flow corridors along actin bundles, neuro-filament arrays, and micro-tubule associated proteins. These fields direct the movement of metabolites and influence the shape of Ca^2+^ micro-domains and the kinetics of soluble signaling complexes [73].

Organelle arrangement contributes to additional hydrodynamic structure. Reconstructions of flow fields created by PINNs have shown that mitochondrial clusters function as hydrodynamic barriers that generate localized vortices at a scale of 20–80 nm. Vortices generated by the hydrodynamic barriers influence the movement of vesicles, lysosomes, and RNA granules, and modulate the timing of trafficking events involved in synaptic remodeling and stress responses [74]. Thermal micro-gradients produced by proton transport across the inner mitochondrial membrane influence viscosity and local diffusion coefficients, thus modifying biochemical reactivity [75]. Chemical gradients arise naturally from these hydrodynamic structures. Changes in proton mobility produce micro-pH gradients that modify the activities of kinases, phosphatases, and metabolic enzymes. Sub-nanometer variations in pH influence hydrogen bonding and electrostatic interactions around enzyme active sites, thus modifying reaction velocities and ligand affinities. AI-aided reaction–diffusion solvers have shown that even weak intra-cellular shear modifies the ability of neurons to buffer calcium by redistributing mobile buffers, such as calbindin and calmodulin. Thus mechanical variability influences biochemical heterogeneity on time scales relevant to synaptic integration [76].

### 4.2. Lipid Surface Microflows, Organelle-Driven Fluid Mechanics, and Mechano-Chemical Feedback

Membranes provide a second two-dimensional fluidic system, whose behavior influences the intra-cellular fluidic behavior. Lipid bilayers form surface flow micro-circuits influenced by gradients in curvature, composition, and surface tension. Molecular dynamics simulations combined with AI-enhanced surface hydrodynamic solvers have revealed that Marangoni-like flows occur on membranes during energy fluctuations, oxidative stress or synaptic activity. These surface flows redistribute lipids like PI(4,5)P_2_, GM1-ganglioside, and phosphatidyl-serine, and therefore influence the clustering of ion channels and receptors, independent of changes in protein concentration [77].

BAR domain proteins, F-BAR domain proteins, and I-BAR domain proteins, which are sensitive to curvature, enhance these surface flows by altering the local bending rigidity. This provides a mechano-chemical feedback loop, wherein membrane curvature influences lipid streaming, lipid streaming modifies protein distribution, and protein distribution influences the local hydrodynamic environment. Such feedbacks can modulate the diffusion of syntaxins, SNAP-25, synaptotagmin, and other components of the exocytic machinery, and indirectly influence the probability of neurotransmitter release and short-term plasticity [78]. Organelle-based fluid disturbances enhance these effects. ER tubules, especially those that are part of mitochondria—ER contacts, produce periodic micro-flows that cause localized changes in osmotic pressure and temporary mechanical deformation of adjacent cytoskeletal structures. Mitochondrial division and fusion, regulated by DRP1, MNF1, and MNF2, modify intra-cellular viscosity and redistribute ATP micro-domains, and thus modify the energetic requirements for intra-cellular cytoskeleton rearrangement and vesicular transport [79]. Small differences in ATP:ADP ratio influence myosin–actin interaction, and thus couple intra-cellular flow rheology to the motion of organelles and intra-cellular trafficking. Collectively, these results suggest that intra-cellular fluidic mechanics undergo continuous reorganization. Manifold learning applied to imaging data sets using AI has shown that the topological evolution of flow field is characterized by gradual drift, rather than abrupt transition; thus, early hydrodynamic instability may be expressed as progressive loss of flow field coherence [80]. Such drift will alter the distribution of metabolites and ions, thus affecting energy supply, redox homeostasis and the long-term capacity for signal transmission. Ultimately, these multi-physics disturbances will accumulate over time, causing a shift in the neuron’s position towards less stable regions of the overall state space [81].

### 4.3. Perivascular Hydrodynamics, Extracellular Fluid Architecture, and Multiscale Flow-Field Drift

Intra-cellular fluidic behavior influences molecular interactions while extra-cellular fluid behavior imposes constraints on larger spatial scales. The Extra-Cellular Matrix (ECM) forms a porous visco-elastic medium whose geometry changes depending on glial remodeling, neuronal activity and metabolic states. Nanovelocimetry has shown that narrow extra-cellular pathways generate directionally biased flow paths with hydraulic resistance determined by glycosaminoglycan density, hyaluronan hydration and perineuronal net architecture [82]. Anisotropic flow patterns generated by the extra-cellular matrix influence the spatial distribution of ions, metabolites, and signaling molecules and establish a connection between the micro-architecture of the extra-cellular matrix and the excitability of the neuron [83].

Perivascular spaces introduce rhythms through oscillatory glymphatic flows, which contain not only the low frequency (0.5–5 Hz) components commonly reported, but also higher-order harmonics > 40 Hz, appearing during sleep, sensory stimulation, or metabolic changes. Decomposition of flows with AI assistance has indicated that the higher-order harmonics modulate solute mixing and affect the dispersion of lactate, bicarbonate and redox active molecules [84,85]. Changes in AQP4 localization at astrocyte end-feet influence hydraulic permeability, and thus modify the amplitude and coherence of peri-vascular waves [86]. Oscillations propagate into capillary adjacent spaces and interact with the geometry of the extra-cellular matrix, leading to non-linear Darcy–Navier–Stokes coupling that generates spatially variable flow velocities. Such flows affect oxygen and glucose gradients and modify intra-cellular energy production, thus modifying intra-cellular protein energy landscapes [87]. Thus, tissue level hydro-dynamics indirectly modify local biochemical and thermal variability. Changes in intra-cellular ionic dynamics occur due to the fluidic flows. Reactive–diffusion solvers for multi-ions, with learned non-linear operators, have indicated that small changes in flow velocities will influence the distribution of potassium ions during neuronal activity and affect the buffering load for astrocytes in the intra-cellular space. Changes in intra-cellular ionic composition due to fluidic flows will modify the driving force for sodium ions, calcium ions, and chloride ions, and will influence synaptic integration and action potential generation [88].

Ultimately, the perivascular and extra-cellular fluid flows set boundaries on all electro-dynamic and biochemical processes that have been previously described. Whole-tissue digital twins using AI have shown that the progressive loss of coherence of the perivascular flow field precedes measurable decline in metabolic resilience, even before structural changes, such as swelling and dendritic spine loss become apparent [89,90]. These models (Table 2) suggest a sequence in which hydrodynamic irregularities develop first and modify solute mixing, ionic stability and thermal equilibrium, thus creating a condition that increases the susceptibility of neurons to molecular and electrical perturbations.

Summary Section: We examine here how active intracellular and perivascular motion could be used to modulate neuronal homeostasis based on mechanical characteristics such as the viscosity of fluids (i.e., resistance to flow), the relationship between pressure and the rate of fluid flow, and the ability of astrocytes to remove substance from the interstitium into the bloodstream. The physical mechanisms mentioned above which restrict the movement of metabolic products, ions and soluble proteins and, therefore, limit their concentration, also limit the diffusion rate and chemical reaction rate and, ultimately, solute clearance and extracellular ionic composition in the brain. Because the transport of material is slow, even small chronic disturbances may cause significant deviations in the electrochemical environment surrounding cells over time. As these studies show, fluidic microcircuitry represents another level of stability that will influence the nature and magnitude of long-term changes in neurons.

## 5. A Multiphysics Failure Framework: Coupled Instabilities Across Molecular, Electrical, and Fluidic Domains

### 5.1. The High-Dimensional Stability Manifold of the Neuron

Resilience of neurons could also be described as the ability of their physical behavior (the combination of their molecular, electrical, mechanical and hydrodynamic behaviors) to remain in a stable high dimensional space (defined by all the possible combinations of the states of all the above-mentioned processes). The physical behavior of neurons does not occur in separate domains; rather, each process is interdependent with the others and their combined effects define the shape and the curvature of the space and its dimensionality [97]. Operator learning-based multi-physics reconstructions show that neurons occupy a region of this high dimensional space that is dynamically stabilized, where local gradients control how mechanical deformation, ion movement, protein micro-states, solvent structure, and metabolic flows are mutually reinforcing [98]. Each point of this high dimensional space defines a tensor of the physical parameters that describe the coupled physical behavior of the neuron, including viscosity fields defined by the cytoskeleton architecture, dielectric heterogeneities determined by lipid composition, topologies of ATP and NAD(P)H micro-domains, matrices of directional ionic mobility, localized curvature–tension states on membranes and perivascular pressure-flow harmonics [99].

Even small variations in any of the physical parameters defining each point in the space cause the geometry of the space to vary. Typically, variations in the geometry of the space manifest as changes in local curvature, reduction in the depth of basins that are stabilizing or an increase in areas where energetic fluctuations have a smaller amount of dissipation as they spread. AI-based analysis of the manifold shows that the neuron moves toward a region of the space where there is less curvature of the dynamics—a region where the gradients of stabilization are flat and the duration that perturbations stay away from equilibrium is longer than expected by the physiological constraints [100]. Therefore, neuronal susceptibility is not the result of discrete biochemical or electrophysiological failures but rather a global deformation of a multi-scale physical landscape. When the neuron is increasingly located in regions of the space where the stabilizing mechanisms do not counteract the cumulative displacement of the various physical fields, then it becomes susceptible [101].

### 5.2. Cross-Domain Coupling and the Emergence of Instability Modes

As the neuron moves around the manifold, synchronized distortions begin to develop in the domains that normally function in synchronization. These distortions lead to the emergence of instability modes—coherent distortion patterns that are indicative of cross-domain divergence and typically first appear early and spread slowly. Current digital twin reconstructions of mechanically, electrically and metabolically simulated field behaviors of neurons indicate several instability modes that develop because of mismatches in time or space between processes that normally work together [102,103]. For example, metabolic–hydrodynamic desynchronization arises when the ATP micro-domains produced by mitochondria are no longer correlated with the nanoscale flow fields within cells. Disruption of the alignment of the micro-domains and the flow fields creates an intermittency in the mismatch between energy availability and the mechanical demand for actin networks, creating slight variations in the patterns of viscous dissipation and modifying the local mechanical relaxation time [104].

Another type of instability mode—dielectric–mechanical divergence—arises when the gradual changes in the lipid order or curvature create a separation between the dielectric relaxation behavior of membranes and the mechanical responses of membranes. Separation of the two types of responses generates small electromechanical delays, creating modest but persistent deviations in the dynamics of channel gating, caused by the lack of coordination between voltage-dependent processes and the mechanical behavior of membranes [105]. Larger scale instability modes include hydrodynamic-ionic phase drift, which arises when the oscillatory perivascular flow loses harmonic coherence [106]. Loss of coherence disrupts solute diffusion and generates stationary non-stationary extracellular ionic gradients, thereby disrupting the long term mean values of the ionic driving forces. A final instability mode—viscoelastic-electrical incoherence—develops when changes in the cytoplasmic rheology affect the way that reaction–diffusion fronts travel through the cytoplasm, thereby changing the spatial and temporal relationships between Ca^2+^ signals and local voltage fluctuations [107]. Instability modes should be thought of as perturbation vectors that displace the neuron in the direction of the manifold’s curvature. Their effects add up: none of them produce obvious pathologic effects alone, but together they alter the physical landscape supporting the neuron’s physiological resilience in such a way that the curvature of the manifold decreases in the direction of the instability modes. As a result, the restoring forces acting against perturbations decrease and small long-lived deviations build-up at all scales [108].

### 5.3. Predicting Collapse Through Multiphysics Bifurcation Dynamics

The progression toward neuronal failure appears to occur along a trajectory through a bifurcation corridor—a narrow strip of state space where the stabilizing forces of the manifold decrease enough so that the coupled displacements become mutually reinforcing. Within this corridor, several indicators of impending failure become apparent through the application of traditional molecular or electrophysiological measurements but are readily apparent when the neuron is considered as a coupled physical system [109]. One of the earliest indicators of failure is the loss of ergodicity in metabolic-mechanical fluctuations. Healthy neurons cycle through a predictable set of states for their ATP micro-domain boundaries, cytoskeletal tension patterns, and viscosity fields. Vulnerable neurons lose ergodicity in these cycles, indicating that the system is not sampling the stabilizing configurations [110].

A second indicator of impending failure is the narrowing of the dissipative frequency band. Healthy neurons absorb disturbances over a wide range of frequencies through mechanical relaxation, ionic buffering, and channel stochasticity. As the manifold flattens, low frequency dissipation weakens because the coherence of hydrodynamics decreases, while the irregularity of high frequency dissipation increases because the sensitivity of stochastic gating noise to dielectric drift increases. Digital twins show that a third indicator of impending failure—distortion of cross-domain covariance—develops when the relationships between hydrodynamic stability, ionic gradients, membrane excitability, and metabolic oscillations become unreliable [111]. Development of this distortion signifies that the sub-systems of the neuron no longer predict each other’s dynamics and therefore represent cross-domain decoupling. Persistent homology of the trajectories of the neuron in high-dimensional space demonstrates that the attractor basins of the neuron fragment into many shallow micro-basins instead of remaining as a single attractor basin. The neuron cycles among the micro-basins, each representing a partially degenerate configuration of multiphysics interactions [112,113]. Fragmentation of the attractor basins signifies a transition from a regime where the neuron remains resilient to a regime where even minor perturbations could send the system into irreversible failure [74]. The multiphysics bifurcation framework provides a unified explanation of why slow and subtle drifts in cross-domain behaviors eventually produce rapid collapse in the late stage of failure. It provides a basis for the use of digital twins as predictive tools by providing a means to identify the deformation patterns of the stability manifold and the early signs of instability modes [114]. The next section will discuss the development of AI-based digital twins as tools for prediction and intervention.

## 6. AI Multiphysics Digital Twins for Forecasting Neuronal Failure Trajectories

### 6.1. Digital Twins as Co-Evolving Representations of Neuronal Physics

Digital twins constructed with AI capabilities can evolve with the living neuron by including multiple types of data (electrical, mechanical, structural, metabolic and hydrodynamic) into a predictive framework. Unlike traditional computational models of neuronal behavior, which use prespecified differential equations to model the behavior of separate systems, digital twins constructed with AI capabilities use cross-domain operators to learn how the concurrent states of all the physical fields influence the subsequent evolution of those fields [115,116].

Experimentally, digital twins may be constructed at multiple organizational levels. These include (a) single-neuron cultures grown on high-density electrode arrays, (b) organoids observed through light-sheet microscopy, (c) brain slices observed with multiphoton voltage sensors, and (d) in vivo preparations combining chronic electrophysiology and genetically encoded reporters [117]. All of these experimental platforms produce data that can be collected and combined to create a common latent representation of the evolving behavior of the physical fields describing neuronal behavior. Because digital twins assimilate asynchronously produced, heterogeneous data streams produced by the aforementioned experimental platforms, digital twins can monitor the evolution of the physical fields describing neuronal behavior under both controlled perturbation and disease relevant conditions [118]. Recent developments in architectures for operator learning enable digital twins to approximate complex, non-linear, multiphysics couplings without requiring explicit specification of the underlying mechanisms. Architectures for operator learning include Fourier Neural Operators, Graph Neural PDE Solvers, DeepONets, Learned Green’s Functions, and Neural Laplace Transform Models. These architectures assimilate time-series data from imaging, nanoscale voltage recording, cytoplasmic nanorheology, metabolic sensing, and extracellular flow measurement to create and continually update a representation of the neuron’s trajectory through the high-dimensional stability manifold described above [119,120]. A number of the architectures described above can be expressed in a probabilistic form (Bayesian Neural Operators and Ensemble-Based Uncertainty Quantification), which enables digital twins not only to predict the future states of multiphysics fields, but also to predict the uncertainty of those predictions. This is particularly important in the vicinity of bifurcation points, since small errors in predicting operators can lead to large differences in the predicted trajectory of the neuron [121].

A key advantage of digital twins is their ability to predict slow drifts in the curvature of the manifold—i.e., slow changes in the stabilizing gradients of the manifold that occur over weeks or months. Instability in multiphysics systems arises from cumulative effects between the physical domains, rather than from discrete events; therefore, the main purpose of the digital twin is not to simulate, but to describe how the neuron moves in state space [122]. Furthermore, through online meta-learning and adaptive refinement of the operators, the digital twin adjusts its own parameters to reflect how the physical domains adapt together, generating a dynamic map that evolves along with the neuron. Thus, digital twins can function as a tool to detect early deviations from stable configurations before the onset of electrophysiological or structural pathology [123].

### 6.2. Learning Across Scales: From Quantum Potentials to Network-Level Field Evolution

In addition to providing an ability to integrate processes occurring at more than ten orders of magnitude in scale, AI-driven digital twins offer a unique ability to map the relationships between processes occurring at multiple scales [124].

At the molecular level, quantum-informed machine learning potentials (e.g., equivariant graph networks, neural wavefunction approximators, and hybrid QM/ML solvers) calculate changes in the potential energy surfaces, the solvation shell polarizability, the morphology of LLPS, and the proton mobility within nano-confined regions. If these models reveal that there are significant changes in the energy gradients surrounding aggregation-prone motifs or membrane-adjacent residues, the digital twin includes these as localized changes in the kinetic rates, thermal fluctuation spectra, and microscopic solvent dynamics [125]. At the mesoscopic level, viscoelastic–hydrodynamic field solvers use physics-informed neural networks to reproduce intracellular velocity fields, viscosity tensors, pressure gradients, and Ca^2+^/pH microdomain geometries. These solvers combine data from particle-tracking velocimetry, ATP-FRET imaging, and mitochondrial thermometry to determine how metabolic flux and cytoskeletal tension affect the movement of materials within cells [126]. The twin learns how viscosity drift, flow incoherence, and mechanical relaxation mismatches affect the distribution of metabolites and energetic constraints [127].

At the cellular and tissue levels, learned electrodynamic operators derived from high-density recordings and ion sensor imaging predict the extracellular impedance fields, ephaptic coupling strengths, nonlinear multi-ion electrodiffusion, and frequency-dependent field propagation. Perivascular flow dynamics are modeled through learned Navier–Stokes operators, which break down glymphatic oscillations into low-frequency transport components and high-frequency harmonics that modify solute mixing. The multiscale components of the digital twin are integrated into graph-based multiphysics co-simulation engines [128,129]. The nodes in these graphs represent physical subsystems and the edges in these graphs represent the learned interactions between these subsystems. By iteratively training the twin, the twin learns how changes in one physical domain (e.g., mitochondrial redox drift, extracellular hydraulic irregularities) affect the evolution of other physical domains (e.g., voltage stability, synaptic responsiveness, hydrodynamic transport efficiency, protein conformational distributions). The integration of the physical domains reveals interaction patterns that cannot be measured through any single experimental method [130].

While the current framework is formulated at the level of a single neuron, analogous operator learning methods can be adapted for populations. When a population is simulated, each neuron in the population is represented by a coupled twin node in a population-level surrogate. In these models, synaptic connectivity, shared extracellular spaces, and common vascular territories are included as additional edges in the co-simulation graph, allowing for the emergence of instability modes that are inherently collective rather than cell-autonomous [131].

### 6.3. Predictive Bifurcation Diagnostics and Intervention-Sensitivity Maps

The ability of AI-based digital twins to predict when and how neurons fail represents perhaps the greatest advantage of this technology. Unlike many previous studies, which have focused on biomarkers indicative of late-stage pathology, AI-based digital twins estimate the temporal geometry of the stability manifold, including estimates of its curvature, contraction, and attractor topology. Several classes of predictive signs of impending instability have been identified recently through research that combines operator learning, spectral decomposition, and topological data analysis [132].

One of the earliest signs of impending instability that the digital twin can detect is loss of ergodicity in fluctuations spanning the physical domains of metabolism, mechanics, and hydrodynamics. The twin detects loss of ergodicity by determining whether time-series of ATP microdomain boundaries, cytoskeletal tension distributions, and intracellular viscosity fields cease to explore a representative sample of their prior state space. Loss of ergodicity indicates that the neuron is still sampling stabilizing portions of the manifold, regardless of whether molecular indicators are within normal limits [133,134]. Another predictive sign of impending instability is the reduction in dissipative bandwidth. The twin calculates how the neuron absorbs perturbations at various frequencies through spectral–energy decomposition. In unstable neurons, low-frequency dissipation is reduced due to reduced cytoplasmic flow coherence, whereas high-frequency dissipation becomes irregular as stochastic gating processes become more sensitive to dielectric and mechanical drift. Reduced dissipative bandwidth is the multiphysics equivalent of reduced physiological resilience [135].

A third sign of impending instability is the degradation of cross-domain covariance, which the twin tracks using learned covariance operators linking hydrodynamics, ionic stability, ATP flux, and electrical excitability. In healthy neurons, these covariances generate stable patterns that maintain functional coherence. As the covariances change, the neuron transitions to a state in which the interactions between the physical fields are no longer mutually reinforcing, indicating the proximity of a bifurcation corridor [136].

Finally, persistent homology applied to high-dimensional state trajectories indicates fragmentation of attractor basins, where the neuron oscillates among weakly separated micro-attractors rather than being confined to a single, dominant attractor. Fragmentation of the attractor basin is one of the clearest mathematical signs of impending failure and the twin can detect it well before structural degeneration has occurred. Verification of the diagnostic signs mentioned here is largely theoretical/computational [137]. Long-term recordings and controlled perturbations of cellular and animal model systems will be required to evaluate the robustness of the diagnostic signs across preparations and whether they depend on specific modeling assumptions. The digital twin can assist in this process by developing hypotheses regarding the trajectory of instability that can then be experimentally tested [138]. The digital twin can also develop maps of intervention sensitivity that quantify the impact of small perturbations in one physical domain on other physical domains. For example, the twin can predict whether restoring glymphatic harmonic coherence, modifying local mechanical relaxation times, or changing intracellular viscosity will restore the curvature of the manifold [139,140].

Therefore, the combination of these properties enables digital twins to transform from descriptive tools into predictive tools that can detect early-phase instability in a multiphysics environment. The digital twin maps the deformation of the curvature of the manifold, describes emergent instability modes, and determines the degree of intervention leverage available at a particular time to either delay or prevent the emergence of a system-wide bifurcation [141]. In summary, AI-driven digital twins represent a new class of AI models that can follow the co-evolving physics of living neurons, rather than the isolated molecular or electrical processes. Due to their ability to incorporate information across multiple scales (quantum, hydrodynamic, metabolic, and network), AI-driven digital twins provide a unified perspective to study how different physical fields drift over time and how such drifts can signal early vulnerability [142].

To develop an operational, multiphysical neuronal failure model, it will be essential to establish a well-organized, collaborative, consortium-based framework, including three key areas: (1) the collection, sharing of data through standardized methods for collecting physical variables and ontologies for those variables; (2) standards for metadata/calibration for data sharing; and (3) distributed, long-term storage systems for large amounts of multimodal time series data (electrophysiology; various ion/molecular sensors; nanorheology; super-resolution microscopy; and perivascular flow). It is unlikely that any single laboratory can collect data from all of the relevant modalities at sufficient temporal resolutions; therefore, development of this model may require multiple laboratories to work collaboratively to generate harmonized, reference datasets for each preparation type (culture–organoid–slice–in vivo) as well as libraries of perturbation types to determine the causal relationships between the coupling coefficients and resultant behavior in the system; as well as libraries of tasks for predicting: (a) drift rates; (b) proximity of bifurcations; and (c) sensitivity of an intervention. In order to accomplish the above, three levels of AI are required: (i) Data Representation/AI-Assisted Data Assimilation (e.g., Self-Supervised Multimodal Encoders; Latent State Space/Neural Stochastic Differential Equation Models); to convert asynchronous, incomplete, or noisy measurements from a variety of instrumentation into a unified latent physical state; (ii) Operator-AI Surrogates (e.g., Fourier Neural Operators; DeepONets; Graph Neural Partial Differential Equations Solvers; Learned Greens Functions/Neural Laplace Operators); to rapidly approximate the cross-domain evolution laws describing the physics of the system; and (iii) Hybrid Mechanistic-AI Inference (e.g., PINNs; Differentiable Programming; Bayesian Neural Operators/Ensembles; Uncertainty Quantification; Causal Discovery); to impose physical constraints; to estimate the values of the coupling parameters; and to make reliable predictions in the vicinity of bifurcations. Barriers to achieving such a model will include: (a) Heterogeneity and Non-Stationarity (Instrument Drift; Adaptation; Batch Effects); (b) Identifiability Across Scales; (c) Sparseness of Ground Truth for Slow Failure Trajectories; (d) High Computational Costs; (e) Uncertainty Due to Distributional Shifts (Species/Preparation/Stage); and (f) Interoperability/Ethics Constraints for Data Sharing—which will require reproducible pipelines; model/version governance; and ongoing, cross-disciplinary collaboration.

The following Table 3 summarizes the principal computational components, physical domains, and predictive capabilities that define current multiphysics digital-twin frameworks.

Summary Section: We propose that digital twin enabled by AI is used as co-evolving descriptions of physical attributes of neurons, and learn the operator functions across many different domains from heterogeneous and time-resolved data. Furthermore, architectures for operator learning and hybrid physics–AI solvers can efficiently approximate multi-physics coupling, and provide forecasting capabilities to predict the long term drift in uncertainty-aware predictions close to bifurcation boundaries. The Digital Twins, therefore, provide a practical computational interface between theoretical frameworks and measurable observations, through representing the common latent space in which all molecular, electrical, mechanical, metabolic and hydrodynamic processes reside. These tools will establish a practical pathway to transform the conceptual framework of our Hypothesis into predictive models.

## 7. Translational and Therapeutic Implications of Multiphysics Digital Twins

### 7.1. Early Diagnostics Through Deformation Signatures of the Neuronal Stability Manifold

The multiphysics paradigm implies that the earliest evidence of deteriorated neuronal function is contained in the changing shape of the neuron’s stability manifold, not in traditional molecular biomarkers. Therefore, the earliest indications of aberrant neuronal health can be detected by analyzing multiscale data (for example, sub-threshold voltage fluctuations, intracellular viscosity spectra, glymphatic harmonic structure, energetic microdomain drift, flow-field coherence, and dielectric relaxation patterns) and comparing them to the expected behaviors of stable attractors using digital twins [151].

Therefore, diagnostics can now be defined as the identification of deformations of the manifold, not of molecular defects. Changes resulting from pathological drifting in the manifold of neuronal stability include:

- Reduced curvature of the manifold’s stabilizing directions;
- Shorter cycles of oscillations in metabolic–mechanical fluctuations;
- Longer dwelling times in unstable saddle areas;
- Reduced synchronicity between intracellular hydrodynamics, extracellular ionic stability, and membrane electromechanical activity.

Therefore, Spectral–topological decompositions of AI architectures can detect these changes long before anatomical or electrophysiological changes occur. Digital twins do not measure single variables; they measure the loss of coherence across the multiphysics domains—an early indication that occurs before aggregation, dendritic atrophy, or metabolic failure [152]. Therefore, this view suggests that future diagnostics may be based on dynamic physics aware biomarkers including intracellular flow entropy, electrodynamic phase coherence coefficients, and cross-domain covariance persistence. Additionally, it may be possible to combine the twin-derived physics biomarkers with the classical molecular biomarkers, where the molecular biomarkers describe biochemical stress while the physics biomarkers describe the physical vulnerabilities caused by the multiphysics interaction [153]. Therefore, a hybrid diagnostic strategy may enable the early differentiation of disease trajectories and differentiate patients with biochemical abnormalities who yet exhibit physical coherence from those in whom both domains drift simultaneously [154].

### 7.2. Mapping Intervention Windows Through Multiphysics Sensitivity Fields

Digital twins provide a mechanism to identify time frames for intervention by providing insight into how perturbations travel through the physically coupled domains of the neuron. Conventional therapeutic strategies usually isolate molecules or pathways to effect treatment, whereas sensitivity field analysis identifies how changes in one domain (hydrodynamic, electrical, metabolic, or mechanical) affect the neuron’s path in the stability manifold [155].

Sensitivity field analysis provides a method to identify the potential leverage points that would otherwise remain invisible. For example, restoring glymphatic pressure harmonics can stabilize the extracellular ionic dispersion and reduce subthreshold voltage drift. Likewise, small adjustments to intracellular viscoelasticity (which can be accomplished by altering the rate of actin cross linker turnover) can re-synchronize the phase relationship between Ca^2+^ micro-domains and local electrical resonance [156]. Additionally, locally adjusting the curvature of membranes via alterations in phospho-inositide distribution can result in uniform dielectric relaxation times that enhance the predictability of gating energetics [25].

Furthermore, digital twins assess the influence of an intervention on the manifold curvature and attractor depth. An intervention that is successful results in increased energetic costs associated with transitioning to unstable states, enhanced ergodicity in metabolic cycles, broadened band-width of dissipation, or enhanced cross-domain covariance. Therefore, in this context, the success of an intervention depends on the restoration of physical stability and not the normalization of individual biomarkers [157]. Since the efficacy of an intervention depends on the neuron’s exact position within its evolving state space, digital twins can produce time-resolved maps of the time frames during which specific types of perturbations have maximal stabilizing effect. These time frames may vary over weeks or months and sensitivity field analysis can determine when hydrodynamic correction is more effective than metabolic realignment, or when membrane electromechanics is the most effective leverage point. These time-resolved maps of intervention time frames can provide guidance for developing adaptive treatment paradigms that evolve in parallel with the patient’s physiological state [158].

### 7.3. Multiphysics-Guided Therapeutic Design: New Classes of Interventions

The multiphysics paradigm serves as a foundation for developing therapeutic strategies that modulate the physical interactions responsible for maintaining neuronal resilience rather than focusing exclusively on end-points of pathology [159]. There are many categories of new therapies that can be developed:

(a) Hydrodynamic Re-coherence Therapies

Therapies that modulate the flow fields within the intracellular or perivascular spaces (by modulating AQP4 localization, fine-tuning of ECM hydration, or regulating actomyosin-driven viscosity) can restore stable solute dispersion and prevent early ionic irregularities [95,160].

(b) Electromechanical Harmonization

Therapies that stabilize curvature–tension relationships on membranes, modulate lipid order, or normalize dielectric relaxation behavior can help to counteract divergence between electrical and mechanical domains [161].

(c) Energetic Phase Synchronization

Therapies that modulate mitochondrial network topology, create a stable architecture of ATP micro-domains, or modulate the amplitude of NAD(P)H oscillations can help to synchronize metabolic flux and hydrodynamic relaxation cycles [162].

(d) Phase State Modulators for LLPS Systems

Agents that modulate phase separation turnover, internal viscosity of droplets, or microstructural ordering can help to prevent mesoscale proteome drift and stabilize conformational landscapes indirectly [163].

(e) AI-Optimized Control Policies

Using reinforcement learning that is constrained by physics laws, digital twins can find the minimum deviation intervention strategies that maximize manifold curvature or minimize instability modes. These learned policies can suggest previously unknown combinations of mild mechanical, thermal, or metabolic modulation that are superior to classical molecular therapies [164].

Collectively, these therapies aim to restore the integrity of multiphysics coupling, rather than reverse damage that had been performed at a later stage. To ensure the safe translation of the digital twin paradigm, reinforcement learning policies can include biophysical feasibility constraints and monotonicity constraints to prevent the algorithm from identifying unphysical or potentially dangerous intervention regimes. Additionally, explainable-AI methods can facilitate an understandable link between learned policies and the underlying neuronal physics [165].

### 7.4. Toward Precision Neurophysics: Clinical Integration of Digital Twin Frameworks

The clinical application of this framework will likely depend on the development of patient-specific digital twins generated from individually collected physiological data such as metabolic imaging, electrical micro-states, sleep-dependent cerebrospinal fluid flow metrics, and individually tailored nanoscale viscosity signatures [166]. By continued assimilation of multiple input modalities, these twins will estimate each patient’s location in the evolving state-space of the neuron and predict vulnerability trajectories months or years prior to diagnosis based on classic criteria [167].

These twins can aid clinicians in guiding real-time therapeutic decision-making by determining:

- When a patient enters a bifurcation corridor;
- What type of intervention(s) will increase the curvature of the stability manifold;
- When metabolic–hydrodynamic ergodicity decreases;
- When there is a trend toward de-synchronization between dielectric-mechanical dynamics;
- And what type(s) of modulation of specific domains will provide the greatest stabilization of the overall system.

**Figure 2:**
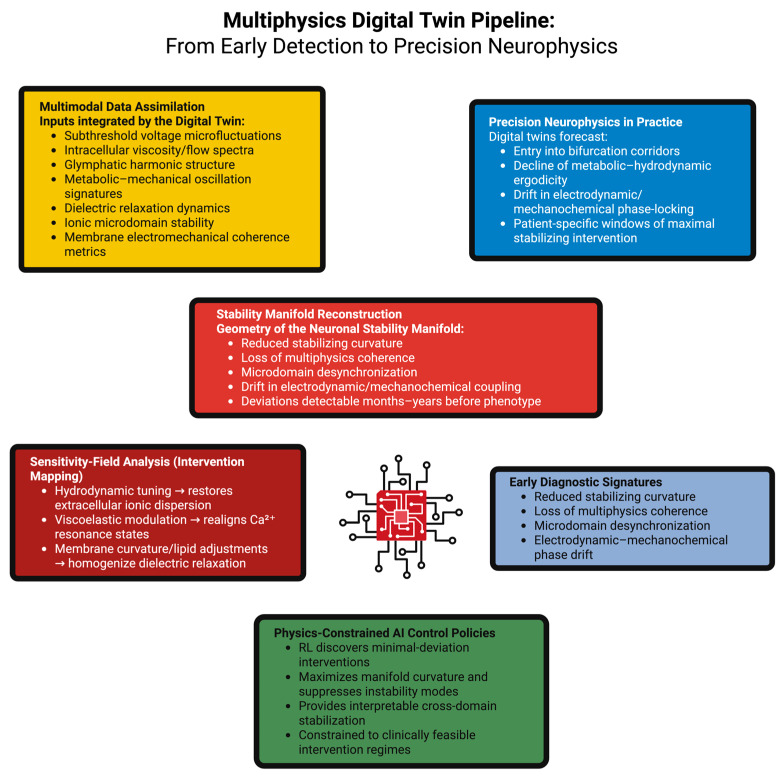
This figure illustrates a multiphysics digital twin framework that integrates heterogeneous neuronal measurements to detect early deviations in stability and guide targeted interventions.

Thus, precision medicine will be re-defined as precision neurophysics: therapy guided by understanding how the entire physical system of the neuron evolves, not by individual molecular measures [168]. As digital twins develop into clinically useful decision support systems, careful attention will need to be given to establishing acceptable bounds on their interpretive authority, protecting patient data, and validating twin-predictions in different biological and demographic populations [169]. Uncertainty quantification and regulatory frameworks adapted to AI-based dynamic models will be important factors in ensuring safe translation. Digital twins integrate disparate physiological signals to reveal early changes in neuronal stability and direct targeted, physics-aware interventions [170]. Figure 2 illustrates this process from data assimilation to manifold reconstruction, early diagnostics, sensitivity mapping, and AI-assisted therapeutic planning.

## 8. Conclusions—Toward a Predictive and Physically Coherent Framework for Neuronal Resilience

Our research has sought to synthesize evidence suggesting that the vulnerability of neurons is caused by the progressive disruption of a multi-domain physical environment; not by individual biochemical or structural abnormalities. Through our prior discussion, we have endeavored to correlate various aspects of the physical environment of neurons (i.e., the dynamics of molecular conformations, the dielectric structure of membranes, the hydrodynamics both inside and outside cells, metabolic flux, and mesoscale electrodynamics) into a single conceptual model in which neurons can only survive when operating in areas of high curvature in a multi-dimensionality-stability manifold.

We proposed a perspective where protein microstates, ionic drifts, ATP microdomains, membrane curvatures and glymphatic flow harmonics were not to be treated individually, but as co-regulated fields whose interaction would result in the long-term resilience of neurons. In doing so, we did not intend to suggest any mechanistic models nor did we want to make any broad generalizations regarding the nature of neuron function, but instead, we wanted to present a conceptual map through which future experimental and computational studies could interpret the initial changes that occur prior to the onset of neurodegenerative disease. Through synthesizing current research in the area of artificial intelligence, particularly operator-learning architectures, physics-informed neural networks, quantum-informed force fields and high-dimensional topological diagnostics, we demonstrated that there exists potential for reconstructing neuronal function across multiple orders of magnitude in terms of scale. These tools allow us to detect deformation signatures in neurons at early time points that remain undetectable using standard analytical methods (loss of ergodicity in metabolic–mechanical fluctuations, narrowing of dissipative bandwidth, or fragmentation of attractor basins in the stability manifold). We emphasized that detecting early deformations of the physical environment of neurons might contain predictive information about subsequent physiological degradation because they represent how all of the physical domains interact in synchrony.

Additionally, we discussed the translational applications of the concept of digital twins of neurons without overemphasizing clinical application. However, the digital twin framework described here is an evolving paradigm rather than a fully developed technology. Nonetheless, it presents a framework in which future diagnostics can measure dynamic resilience rather than static biomarkers, and in which therapeutic interventions can be assessed based on whether they restore curvature and stabilize neurons, rather than reversing damage that has already occurred. It is our hope that this paradigm will encourage the development of therapies that address hydrodynamic coherence, membrane electromechanics, mesoscale rheology and/or energetic-phase alignment, as complementary to molecular therapies, in addressing the physical coupling of neurons that maintains their stability. As this review comes to a close, there are many unanswered questions. The exact geometry of the stability manifold is currently unknown, and likely varies with cell type, developmental stage, network environment, and metabolic state. There is a need for systematic mapping of the pathways through which deformation of one physical domain results in deformation of another physical domain. Finally, designing safe and precise interventions that modulate the physical coupling between neurons without inducing unwanted effects is a major challenge. Rather than presenting a final model, we see this work as an invitation to study these questions using increasingly refined experimental and computational methods.

If the concepts presented here have an effect, it may be to inspire a paradigmatic shift in how we view neurodegeneration as a process; from a series of pathological events to a gradual transition of the physical environment of neurons across a continuously deforming physical environment. Ultimately, we believe that by integrating molecular insights, biomechanical reasoning, electrodynamic modeling, and AI-based multiphysics reconstructions of neuronal systems, future studies should define the first trajectories in which neurons move toward instability and provide the opportunity to alter those trajectories well before irreversible collapse occurs.

Finally, we recognize that the complexity of the physics governing neurons far exceeds what can be addressed in a single review. Our purpose was to bring together the developing threads of a young field that is only just beginning to outline its dimensions, and to arrange them in proximity so that the relationships between them become clearer. We hope that the conceptual framework provided here establishes a foundation for continued investigation and that future studies will enhance, critique and extend these ideas to improve the understanding of neuronal resilience.

Concluding outlook and testable predictions. This model also predicts some empirically testable hypotheses. There will be evidence of “drift” in the strength of connection of vulnerable neurons through all of their multiphysical interactions; i.e., there will be evidence of a measurable reduction in cross-domain covariance of metabolic flux, cytoplasmic rheology, membrane excitability and perivascular clearance prior to apparent aggregation or structural degeneration. Additionally, there should be a predictable decrease in the “dissipative bandwidth” of a neuron’s multi-scale fluctuation dynamics and a loss of ergodic behavior in those same fluctuations. The second predictive hypothesis is that the first signs of instability will occur at the point of convergence of changes across multiple domains; i.e., minor changes in the population of conformational microstates and slight changes in gating and/or dielectric properties and/or incoherence in the transport process of various molecules. The third predictive hypothesis is that interventions to maintain stability in the connections between domains (energy availability, membrane dielectric structure, intracellular viscosity, and harmonic perivascular transport) will have a higher degree of stabilization than interventions targeting only static endpoints. These stabilization effects should be quantifiable through sensitivity maps developed from longitudinal multiphysics recordings. The development of standardized multimodal datasets and standardized perturbation protocols will enable the verification of the above predictive hypotheses and the translation of theoretical models into empirical-based forecasting models (“digital twins”).

## Figures and Tables

**Table 1:** This intends to demonstrate how a series of small changes to the energy levels of proteins (neural) can cascade into large scale, structural phase transitions of neurons by combining cellular, mesoscale, and quantum level views. This is possible for detection using an artificial intelligence-based reconstruction of the free energy and analysis of phase behavior. Abbreviations and symbols: Å, ångström; nm, nanometer; µm, micrometer; AI, artificial intelligence; ANI, Accurate NeurAl networK engINe; QM/ML, quantum mechanics/machine learning hybrid modeling; ΔG‡, activation free energy; kBT, thermal energy (Boltzmann constant × absolute temperature); LLPS, liquid–liquid phase separation; ATP, adenosine triphosphate; ADP, adenosine diphosphate; RNA, ribonucleic acid; α-synuclein, alpha-synuclein; TDP-43, TAR DNA-binding protein 43; FUS, fused in sarcoma; hnRNPA1, heterogeneous nuclear ribonucleoprotein A1; PSD-95, postsynaptic density protein 95; ROS, reactive oxygen species; Hsp70/Hsp90, heat shock protein 70/90; NMR, nuclear magnetic resonance.

Analytical Scale	Key Mechanisms and Physical Principles	Representative Molecular Systems	Functional Consequences and Pathophysiological Relevance	References
Quantum–Molecular (Å–nm)	AI-derived neural network potentials (ANI, QM/ML hybrids) reconstruct protein free-energy surfaces; shallow basins (<10 kJ/mol barriers) permit microstate redistribution under minor physicochemical shifts (pH, ions, redox).	Tau (VQIINK/VQIVYK motifs), α-synuclein (E61–A76 cluster), TDP-43/FUS low-complexity domains.	Microstate drift without large conformational change; early disruption of hydrogen-bond and cation–π networks destabilizes local folding equilibria.	[36]
Mesoscale (nm–µm)	Multivalent sticker–spacer interactions drive LLPS; phosphorylation, methylation, ATP, and RNA modulate phase diagrams; oxidative stress and ionic drift alter droplet viscoelasticity and aging kinetics.	TDP-43, FUS, hnRNPA1 condensates; PSD-95–Shank–Homer synaptic assemblies.	Altered condensate turnover, increased gel fraction, impaired diffusion; early deviation from proteome homeostasis preceding aggregation.	[37]
Cellular–Organellar (µm)	Protonation, redox, and ionic gradients reshape local dielectric constants; erosion of energy barriers (ΔG‡ ↓ ~1–3 kBT) accelerates conformational transitions; mitochondrial potential fluctuations and ROS feedback deform landscapes.	Cytoskeletal proteins, chaperones (Hsp70/Hsp90), respiratory-chain complexes I–IV.	Reduced energy-barrier selectivity and impaired structural maintenance; enhanced conformational noise and ROS propagation.	[38]
Network and Phase-Transition Level	Collective drift across proteome phase boundaries driven by osmotic imbalance, ATP/ADP ratio, and macromolecular crowding; critical-point crossing between liquid-like and gel-like states.	Neuronal proteome viewed as multi-variable phase manifold (ionic composition, condensate valence, solvent polarization).	Emergence of mesoscale rigidity, slowed turnover, altered signal propagation; pre-aggregation instability signature.	[39]
Emergent Biophysical Signature (System)	Reduced curvature and friction on conformational energy surfaces; broadened NMR relaxation dispersion, slower folding kinetics, altered fluorescence lifetime distributions.	Ensemble of neuronal proteins under chronic stress or energy imbalance.	Early physicochemical marker of neuronal vulnerability before morphological pathology or aggregate formation.	[40]

**Table 2:** Summarizes the multi-scale fluidic processes that govern the stability of neurons organized by tiers representing intracellular, membrane associated, extracellular and perivascular compartments. Each Tier describes the major hydrodynamic behaviors, the molecular and biophysical forces that drive them and the functional consequences of drifting flow coherence over time.

Domain	Core Fluidic Phenomena	Mechanistic Drivers & Molecular Contributors	Functional Consequences for Neuronal Stability	References
Intracellular Quantum–Hydrodynamic Layer	Nanoconfined water networks; proton delocalization; shear-sensitive viscoelastic flow	Hydrogen-bond network fluctuations; mitochondrial redox microgradients; actin-mediated shear thinning; proton conduction chains informed by ML-QM potentials	Alters protonation of voltage-sensor residues; shapes Ca^2+^ microdomains; modulates reaction rates and local pH microgradients	[91]
Cytoplasmic Mesoscale Flow Architecture	PINN-reconstructed anisotropic flow corridors; organelle-induced vortices; ATP-dependent viscoelastic rheology	Actin/filamin remodeling; microtubule alignment; mitochondrial clustering; local thermal gradients	Controls metabolite distribution; sets timing of vesicle/RNA granule trafficking; introduces chemical heterogeneity that precedes synaptic dysfunction	[92]
Membrane & Organelle Surface Microcircuits	Marangoni-like lipid flows; curvature-driven surface streaming; mechano-chemical feedback loops	PI(4,5)P_2_, GM1, PS redistribution; BAR-domain curvature sensors; ER–mitochondria Ca^2+^ pulses; Drp1/Mfn-mediated viscosity shifts	Reconfigures ion-channel clustering; modulates exocytic probability; shifts energetic thresholds for cytoskeletal remodeling	[93]
Extracellular Matrix Fluid Networks	ECM as porous viscoelastic conduit; anisotropic nanoflows; activity-driven remodeling	Hyaluronan hydration; glycosaminoglycan density; perineuronal net geometry; astrocytic ECM sculpting	Dictates spatial ion gradients; shapes ephaptic coupling; modifies excitability through constrained diffusion corridors	[94]
Perivascular & Glymphatic Hydrodynamics	Multi-frequency oscillatory flows (0.5–5 Hz, 40–90 Hz harmonics); AQP4-dependent wave coherence; nonlinear Darcy–Navier–Stokes coupling	Astrocytic endfoot polarity; arterial pulsatility; metabolic state; CSF–ISF mixing dynamics	Modulates solute clearance, redox balance, and ion dispersion; governs metabolic resilience and sets boundary conditions for neuronal phase stability	[95]
Whole-Tissue Hydrodynamic Integration	Flow-field drift; loss of coherence across scales; energy–fluid coupling	Decreased AQP4 polarization; ECM stiffening; mitochondrial energy deficits; altered ionic buffering	Early destabilization before morphological change; heightened susceptibility to molecular and electrophysiological perturbations	[96]

**Table 3:** Aims to condense the essential functional aspects in which AI-based multiphysical digital twins simulate and predict neuronal stability.

Functional Axis	Core AI/Computational Mechanisms	Physical Domains Captured	Key Emergent Capabilities	References
1. Co-evolving multiphysics representations	Operator-learning frameworks (FNOs, DeepONets, graph neural PDE solvers); probabilistic neural operators for uncertainty-aware forecasting	Structural, electrical, metabolic, mechanical, hydrodynamic	Continuous assimilation of heterogeneous data; tracking of slow manifold curvature drift; adaptive updating under perturbations	[143]
2. Cross-scale molecular integration	Quantum-informed ML potentials (equivariant networks, neural wavefunction solvers) coupling conformational energy landscapes to reaction–diffusion operators	Conformational energetics, LLPS microstructure, solvation-shell polarity, proton mobility	Early detection of shifts in aggregation-prone motifs; mapping molecular instability into mesoscale reaction kinetics	[144]
3. Mesoscale hydrodynamic–viscoelastic solvers	Physics-informed neural networks (PINNs) for intracellular flows, viscosity tensors, pressure fields	Intracellular rheology, Ca^2+^ microdomains, metabolic flux fields	Identification of viscosity drift, flow incoherence, mechanical relaxation mismatch; linking cytoplasmic mechanics to energetic constraints	[145]
4. Electrodynamic field reconstruction	Learned electrodiffusion and impedance operators; neural Green’s-function approximators	Extracellular ionic dynamics, ephaptic fields, nonlinear multi-ion transport	Detection of irregular frequency-dependent dissipation; mapping of electrodynamic fragility preceding firing instability	[146]
5. Perivascular/glymphatic flow modeling	AI-enhanced Navier–Stokes solvers; harmonic decomposition of vascular oscillations	Perivascular fluid architecture, AQP4-dependent permeability, solute mixing	Linking flow-field coherence to metabolic resilience; identifying high-frequency harmonic loss as early instability marker	[147]
6. Multiscale co-simulation graphs	Graph-based multiphysics co-simulation engines integrating coupled operator nodes	Organellar, cellular, tissue, and microvascular subsystems	Revealing cross-domain coupling not visible experimentally; emergence of collective instability modes	[148]
7. Early-phase bifurcation diagnostics	Spectral-energy decomposition, manifold learning, topological data analysis (persistent homology)	Stability curvature, attractor topology, ergodicity metrics	Detection of dissipative bandwidth narrowing, covariance breakdown, attractor fragmentation prior to structural pathology	[149]
8. Intervention-sensitivity and control maps	Neural sensitivity fields; differentiable multiphysics surrogates enabling counterfactual simulations	Mechanical, hydrodynamic, metabolic, electrodynamic	Forecasting intervention windows; estimating how local perturbations propagate across physical domains to restore stability	[150]

## Data Availability

No new data were created or analyzed in this study. Data sharing is not applicable to this article.
